# Transgenerational effects and the cost of ant tending in aphids

**DOI:** 10.1007/s00442-013-2659-y

**Published:** 2013-05-21

**Authors:** Karolina Tegelaar, Robert Glinwood, Jan Pettersson, Olof Leimar

**Affiliations:** 1 Department of Zoology, Stockholm University, 10691 Stockholm, Sweden; 2 Department of Ecology, Swedish University of Agricultural Sciences, 75007 Uppsala, Sweden; 3 Wissenschaftskolleg zu Berlin, Wallotstrasse 19, 14193 Berlin, Germany

**Keywords:** Aphid–ant mutualism, Maternal effects, Reproductive investment, Embryo size, Plasticity

## Abstract

In mutualistic interactions, partners obtain a net benefit, but there may also be costs associated with the provision of benefits for a partner. The question of whether aphids suffer such costs when attended by ants has been raised in previous work. Transgenerational effects, where offspring phenotypes are adjusted based on maternal influences, could be important in the mutualistic interaction between aphids and ants, in particular because aphids have telescoping generations where two offspring generations can be present in a mature aphid. We investigated the immediate and transgenerational influence of ant tending on aphid life history and reproduction by observing the interaction between the facultative myrmecophile *Aphis fabae* and the ant *Lasius niger* over 13 aphid generations in the laboratory. We found that the effect of ant tending changes dynamically over successive aphid generations after the start of tending. Initially, total aphid colony weight, aphid adult weight and aphid embryo size decreased compared with untended aphids, consistent with a cost of ant association, but these differences disappeared within four generations of interaction. We conclude that transgenerational effects are important in the aphid–ant interactions and that the costs for aphids of being tended by ants can vary over generations.

**Electronic supplementary material:**

The online version of this article (doi:10.1007/s00442-013-2659-y) contains supplementary material, which is available to authorized users.

## Introduction

Mutualism is often described as an interaction between species that benefits all participants (Boucher et al. 1982) and where each organism increases its fitness by utilizing services of the partner species. Several types of mutualism are recognised, with varying levels of engagement, ranging from by-product mutualism where there are no investments, through pseudo-reciprocity (Connor 1995), where one partner invests, to mutual pseudo-reciprocity (Leimar and Connor 2003; Leimar and Hammerstein 2010) and reciprocity (Trivers 1971; Axelrod and Hamilton 1981), where there is two-sided investment. More broadly, to varying degrees, cooperation and conflict both play a part in supposedly mutualistic interactions (Herre et al. 1999), also involving phenomena such as punishment, sanctions, and coercion (Raihani et al. 2012).

A division of mutualistic interactions into categories is important because investing resources is costly and is only expected to occur if it increases the benefit received by the investing organism from its partners. The presence of costs suggests, therefore, that the investment is an adaptation to mutualism. In general, the question of costs of mutualism has long been part of the study of the ecological dynamics of these interactions, and the impression is that such costs can vary considerably between systems and over time and space (Bronstein 2001). Our aim here is to investigate experimentally whether aphids incur investment costs in their interaction with ants.

Aphid honeydew is produced as a by-product of phloem sap feeding, and constitutes a nutrient-rich food source for foraging ants. It has been claimed that the interaction between ants and aphids is a win–win game where both participants benefit without incurring costs (El Ziady and Kennedy 1956; Banks 1958; El-Ziady 1960). Because honeydew is also produced in the absence of ants, these early studies argued that the aphids invest nothing in the interaction and benefit from the protection afforded against parasitoids and predators, and from the removal of the sugary residue. This theory was subsequently revised (Stadler and Dixon 2005), because studies indicated that there can be costs for the aphids in the interaction. For instance, Stadler and Dixon (1998) showed experimentally that aphids suffered costs in the form of slower development and reduced colony growth when investing in, or interacting with, ants. It has also been found that aphids are able to alter the honeydew composition and droplet delivery rate when tended by ants (Yao et al. 2000; Fischer and Shingleton 2001; Yao and Akimoto 2001, 2002). Such alterations could be costly for the aphids and signal that an investment is made by the aphids. The initial contact between aphids and ants could have a special importance in this regard, because of the need for aphids to avoid ant predation and to establish the ant association. There is thus reason to expect a higher aphid investment in the initial phase of the interaction (Glinwood et al. 2003; Endo and Itino 2012).

The removal of honeydew residue by ants is advantageous for the aphids because they can get trapped in the sticky substance. The residue can be a substrate for fungal growth (Dik et al. 1991; Pike et al. 2002) and serve as a cue for parasitoids, thus reducing the quality of the aphid environment (Budenberg 1990). The photosynthetic capacity of the plant can also be reduced by the honeydew cover of the leaf and the sooty mold growth (Vereijken 1979; Rabbinge et al. 1984).

The ant–aphid interaction has been well studied under both field and laboratory conditions (e.g., El Ziady and Kennedy 1956; Banks 1958; El-Ziady 1960; Breton and Addicott 1992; Stadler and Dixon 1998; Flatt and Weisser 2000; Yao and Akimoto 2002). Most experiments have been conducted over relatively short periods of a single or a few aphid generations, although Stadler and Dixon (1998) examined four successive generations. Here, we study the ant–aphid interaction over a longer period of time, including varying the presence of ants over a total of 13 aphid generations, in order to be able to examine transgenerational effects of ant tending. Currently, the phenomenon of transgenerational as opposed to within-generation plasticity is receiving considerable attention (Bonduriansky and Day 2011), although maternally-induced differences in aphids have long been noted as an important cause of phenotypic variation (e.g., McKay and Wellington 1977).

Parthenogenetic aphid females are not restricted by fertilization, which is a reason to expect transgenerational effects. Aphid ovarioles can contain up to two successive generations of developing offspring, and this telescoping of generations may allow offspring to be influenced by the maternal investment into embryos. In aphid reproduction, the trade-off between number and size of offspring is partly determined by the size-distribution of the embryos in the aphid ovarioles. A large number of small embryos results in a high rate of offspring production, whereas more varied sizes of the embryos, with a sharper increase in size towards the ovariole posterior, can result in fewer but larger offspring (Dixon and Dharma 1980a). Transgenerational effects are generally thought to be important in several aspects of aphid biology, including in the production of alates (Dixon 1998; Müller et al. 2001; Braendle et al. 2006), in connection with ant attendance (El-Ziady 1960) and in the determination of other aspects of offspring phenotype (Mondor et al. 2008).

Aphids cannot easily terminate a less profitable interaction with ants, but they may be able to vary the level of investment. As is the case for many mutualisms, one should expect interactions between aphids and ants to be dynamic in response to conditions that vary in space and time (Bronstein 1994). The prediction emerges that aphids should adjust their investments in ant rewards, both in response to their need for protection from enemies and to the willingness of ants to remain in attendance after an interaction has started. In situations where aphids assess the risk of attack by predators and parasitoids as relatively low, as could be the case in our laboratory set-up, one possible outcome is that aphids initially invest more heavily in providing benefits for ants, in order to ensure a sustained ant foraging response, but then gradually decrease their investment, perhaps over successive generations. This kind of temporal pattern of investment has previously been observed for the mutualistic interaction between lycaenid larvae and ants (Axén et al. 1996). There are in fact a number of studies showing that the degree of investment in ant rewards by trophobionts is flexible and can respond to changes in the perceived risk of enemy attack (Leimar and Axén 1993; Axén et al. 1996; Axén and Pierce 1998; Agrawal and Fordyce 2000; Morales et al. 2008). This kind of flexibility might also be present in aphids and might involve transgenerational effects. Our study is the first attempt at investigating this possibility.

## Materials and methods

### Study species

To study the effect of ant tending on aphid growth and reproduction, we used the black bean aphid *Aphis fabae* (Scopoli 1763) (Homoptera: Aphididae) and the black garden ant *Lasius niger* (Linné 1758) (Formicidae: Formicinae). *Aphis fabae* is facultatively myrmecophilous (Stadler and Dixon 1998), heteroecious, and polyphagous, with a wide range of secondary hosts (Blackman and Eastop 2000). The aphids were derived from a monoclonal colony originating from the UK (Rothamstead Research) and were reared on broad bean (*Vicia faba* ‘Hangdown Grünkernig’), a secondary host plant used by *A. fabae* in the parthenogenetic summer cycle. *Vicia faba* has nectaries where ants are able to forage for nectar.

Bean seeds were soaked in water for 1 day before planting and were planted 12–15 days before use. The plants in the paired cages (see below) were similar in height (13.5 ± 1.8 cm) and no significant difference in plant growth within these pairs were found. The same type of soil (“Plantagen: Blomjord med leca”) was used for all plants, and they were watered daily through a watering tube in the side of each cylinder.

**Fig. 1:**
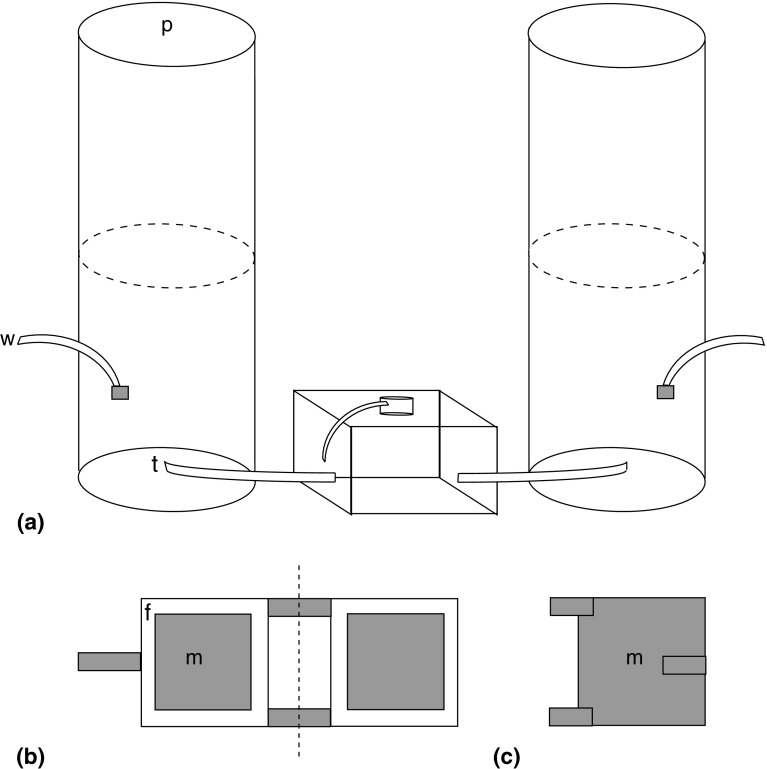
**a** Experimental set-up. A cage pair consisted of two Plexiglas cylinders 40 cm high, that could be taken apart at 20 cm (*dashed line*) and were sealed in the bottom and connected to an ant colony with a sealable tap (*t*) to allow changes in treatment. The cylinders each had a watering tube (*w*) leading to the bean plant pot, to minimize daily disturbance of the black bean aphids *Aphis fabae* on the plant. A perforated sheet of plastic (*p*), allowing light intake and air circulation, covered the top of each cylinder. Black garden ant *Lasius niger* colonies had access to a feeding arena at the top of the nest. Open (**b**) and sealed (**c**) clip cages were made from rubber foam (*f*) and fine metal mesh (*m*). *Dashed line* indicates where hinges fold

*Lasius niger* has previously been shown to tend *A. fabae* (El Ziady and Kennedy 1956; Banks 1958; Stadler and Dixon 1998; Offenberg 2001; Fischer et al. 2005) and is found in various habitats, both dry and damp (Zahradnik 1991). Nests were dug up at Frescati, Stockholm, and each laboratory colony originated from a separate nest. All laboratory colonies contained ant brood and were queenless. They were kept in nest boxes (16 × 16 × 11 cm) connected to a separate feeding arena (7.5 cm Ø; see Fig. 1), where they were fed Bhatkar diet (Bhatkar and Whitcomb 1970) every fourth day (see Leimar and Axén 1993 for similar ant maintenance). There were 12 laboratory colonies in nest boxes and each contained soil and several cotton-plugged test tubes filled with water.

### Experimental set-up

The experiment was conducted from 16 September to 19 December 2008, at Stockholm University, Sweden, in a laboratory space with no access to natural daylight. The room temperature was 24.4 °C ± 0.35 (mean ± SD) and the photoperiod regime was 22:2 h light:dark, in order to maintain the aphids in a parthenogenetic summer cycle.

The interaction between aphids and ants was studied in an experimental system especially constructed for this purpose. The system consisted of 24 cylinders paired in 12 experimental units. Each unit was made up of two vertically positioned Plexiglas cylinders (cages) connected to an ant nest box with PVC tubes that had closable valves to permit changes in ant tending (Fig. 1a). Cages were paired in order to allow ant access to only one of the cages in a pair, with the other acting as a control. The two cages in each pair were randomly labeled as A and B.

The cylindrical cages had a diameter of 21 cm and a height of 40 cm, and it was possible to remove the upper half to place or remove bean plants and aphids (Fig. 1a). A perforated plastic foil was used to cover the top of the cage to maximize light transmission for plants, and the bottom of the cage was made of a plate of Plexiglas. The upper part of each cylindrical cage and each ant nest box had a 3-cm broad-layer of Fluon^®^ (Northern Products, Woonsocket, RI, USA) to prevent aphids and ants from escaping. A 4-mm-Ø PVC tube was inserted though the lower part of each cage so plants could be watered with minimal disturbance to the aphid colony. No aphids or ants could escape through the tube. Each cage was placed under two florescent lamps; one Osram 18W/21-810 and one Sylvania Gro-LuxF18W/Gro-T8 (Ton et al. 2007), with two aluminium reflectors to mimic natural sunlight, resulting in a light intensity of 1,623 ± 75 lx over the cylinders.

Small clip cages (3 × 3 cm) made of fine metallic mesh and foam rubber were used to enclose groups of aphids on plants at the start of each experimental generation; this was done to increase the likelihood that aphids stayed together in a group (Fig. 1b). Aphids were handled with a fine brush.

### Data collection

A milligram scale (Cahn 28 automatic electrobalance; Cahn instruments) was placed in the same room as the experimental units, in order to weigh aphids without exposure to natural daylight. Aphids were weighed alive and in groups of ten in a standardized procedure in small cups where they were not able to escape because of fluon^®^-coated walls.

All aphids except the four founders of the next generation were preserved in 70 % ethanol. Five adult aphids from each cylinder from generations 2, 3, and 6 were dissected and their ovarioles removed. After 7 days (or longer) in 70 % ethanol, all embryos were dark in color and could be counted and grouped into size categories using a microscope. For dissection, fine tweezers were used to first remove the head of the aphid and then open the back shield from the neck to the cauda and remove the ovariole package. The embryos were spread out for counting and assignment to size categories (large embryo: length >0.05 mm; medium: between 0.03 and 0.05 mm; small: between 0.005 and 0.03 mm), using a 0.01-mm graticule slide (see Fig. S1, online resource 1, for images of ovarioles).

As a separate procedure, aphids were reared in low- and high-density colonies, in order to examine the relationship between dry weight and wet weight (all other aphid weights in this study are wet weights). The adult aphids were first euthanized by freezing at −20 °C for 48 h. Their wet weight was determined for groups of ten aphids in small aluminium baskets. Aphids were then dried in the baskets for 48 h in 60 °C, and weighed on the same scale that was used for wet weights. During the process, fine tweezers were used to handle the baskets to minimize transfer of moisture.

### Experimental procedure

The experiment was conducted over 13 weeks, with a new generation of aphids produced each week. A 4-week tending regime was used, as in Stadler and Dixon (1998). During the first two generations, all cages were untended; in generations 3–6, cage A of each pair was ant tended (first trial); in generations 7–9, all cages were untended; and in generations 10–13, cage B of each pair was ant tended (second trial; reversal of the ant treatment in the first trial).

At the start of the experiment, and for each successive week, four adult aphids were used to found a colony in each cylinder. From the second week onwards, the founders in a given cylinder were chosen from among the newly molted adult offspring of the founders of the previous generation in that cylinder. Each founder was weighed individually. For the start of the experiment, the total weight difference between the founding aphid colonies in a pair of cages was kept to a minimum (and was at most 0.036 mg). For the founding of the next and all subsequent generations in each cage, individuals were chosen to represent the size range of adult aphids present in that cage in the most recent generation. If there were not enough founders to start a new generation for both cages of a pair, the experimental unit was terminated at that time. Of the 12 original pairs, 11 were used in the first trial (generations 3–6) and nine in the second (generations 10–13).

The four founding aphids were placed in a clip cage on a fresh bean plant and left for 24 h. In each pair, bean plants were chosen to match in size. After 24 h, clip cages were removed and the four aphids were left for 6 days in the cage, either with or without ant access. After 6 days, the bean plant was cut at the base, and plant height, total adult aphid weight, number of adults, and total nymph weight were recorded.

The bean plants were measured from the base of the stem to the top leaf at the start of each experimental week, the following day (before removing the clip cage), and after cutting the stem on day 7. The number of ants on each bean plant was counted after cutting the plant and the ants were then returned to their nest. The presence of fungus on the leaves of the plant was also recorded.

### Statistical analysis

The data on aphid weights as a function of ant treatment and time after start of ant attendance were analyzed with Bayesian methods: linear mixed models were fitted using the MCMCglmm function (v.3.17; Hadfield 2010) in the R statistics package (v.2.15.2; R Development Core Team 2012). The R package was also used for all other statistical tests. See online resource 2 for the R code used and datadryad.org for the data files (doi:10.5061/dryad.s4s2b).

As response variables *y*, we used differences between the tended and untended cage in a pair at the end of a generation. For each of the two trials, we included generations from the one before the start of ant treatment up to the fourth generation of ant treatment in the trial. The quantities we examined were differences in total colony weight, average adult weight, and total nymph weight. As an example, for total weight there was one observation (*y*) of the difference in weight between the tended and untended cylinder in a pair for each included generation. We examined how these differences depended on the number (*T*) of generations after the start of the ant-tending treatment, with *T* = 1 corresponding to the first generation of ant tending, which was generation 3 for the first trial and generation 10 for the second trial. We fitted relationships like

1 $$ y = a + b\left( {T - T_{0} } \right) + c\left( {T - T_{0} } \right)^{ 2} +\,{\text{residual}} $$

where *a* is an intercept, *b* a slope, *c* a coefficient of a quadratic term, and *T*_0_ = 2 was used to center time around the second generation after the start of ant tending. This means that the intercept *a* is the effect of ant tending at the end of the second generation of ant attendance, and *b* and *c* express a time-dependence of the effect. The experimental design provided data for *T* = 0, 1, 2, 3, and 4 for a total of 20 observational units (*T* = 0 is the generation immediately before the start of ant tending; there were 11 cage pairs in the first trial and 9 cage pairs in the second, although, for a few units, observations at the end of the period of ant attendance were missing).

The aim of the statistical analysis was to estimate and test the parameters *a*, *b*, and *c* in Eq. (1) as fixed effects. We used the posterior mean values as estimates. By fitting linear mixed models, we also estimated random effects, at the level of the observational unit (a cage pair in a trial), for some or all of these parameters. These random effects could, for instance, correspond to differing intensities of ant attendance in different cages. Our reason for using a Bayesian Markov Chain Monte Carlo method is that this is a reasonable approach to assess the statistical significance of fixed-effect parameters in models with random effects. We report the Bayesian 95 % credible intervals (highest posterior probability density intervals) for the parameters *a*, *b*, and *c*, together with a related MCMC *p* value provided by the MCMCglmm function. We used the Deviance Information Criterion (DIC; Spiegelhalter et al. 2002) to assess which random effects to include in a model. This criterion is a generalization of the AIC, which is computed in Bayesian MCMC analysis and which can be used for model selection. Just as for AIC, a smaller value of DIC indicates a better-fitting model.

To analyse models corresponding to Eq. (1) with the MCMCglmm function, we used 10,000 burn-in iterations, followed by 250,000 iterations sampled with a thinning interval of 25, resulting in a sample size from the posterior distribution of 10,000. The variance components of the random effects were given inverse-Wishart prior distributions with variance parameters such that the total observed variance in *y* was split evenly between the residual and the random effects, and if there was more than one random effect, the prior gave on average equal weight to each of them.

In addition to the analyses of aphid weights, we also examined aphid embryo sizes. For the statistical analysis of the effect of ant tending on the distribution of embryo sizes, we used a multivariate response variable given by

$$ (log(y_{\text{L}} + 1),\;log(y_{\text{M}} + 1),\;log(y_{\text{S}} + 1)) $$

where $y_{\text{L}}$, $y_{\text{M}}$, and $y_{\text{S}}$ is the number of large, medium-sized, and small embryos in a dissected adult aphid (we dissected 5 adult aphids per cage). The log-transformation made the response variables approximately normally distributed. We used this trivariate variable as response in MCMCglmm model fitting, with ant treatment as fixed effect and the cage pair as random effect. We performed two separate such analyses of the effect of ant tending, one for generation 3 (the first ant-tended generation) and one for generation 6 (for which there had been ant tending in the current and the three previous generations). Our aim for choosing these analyses was to examine the effect of ant tending on embryo size distribution both in an early phase of ant tending and after several generations of ant tending. Finally, we examined whether there were changes between generations 2, 3, and 6 in the embryo size distribution in untended aphids, again using the above trivariate response variable in MCMCglmm model fitting, with generation as fixed effect and cage as random effect.

## Results

### Wet weight and dry weight

**Fig. 2:**
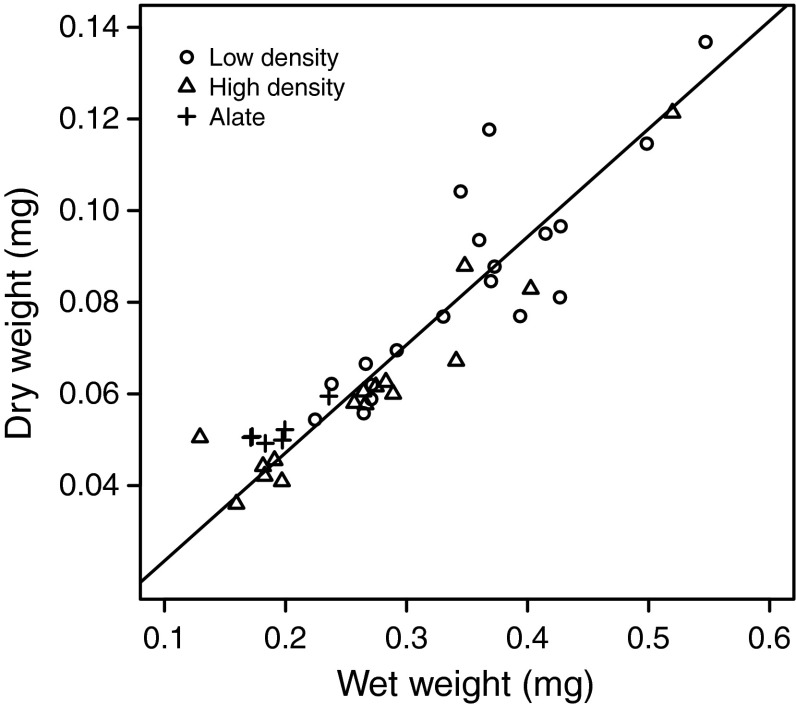
Relation between dry and wet weight in *A. fabae* adults reared on bean plants. Aphids used were from either high-density colonies (*triangles*) or low-density colonies (*circles*). Winged individuals (alate) were from high density colonies. Each* data point* is the mean of ca. 10 individual aphids. The *line* is a regression through the origin of dry weight on wet weight with a common slope

Statistical analysis indicated that a very simple statistical model, in the form of a linear regression through the origin of adult aphid dry weight on wet weight, gave the best fit (measured using AIC), compared with models with different intercepts and/or slopes for the different categories of aphids. The fit to data of this simple model was quite good, with a coefficient of determination (*R* ^2^) of 0.98 (Fig. 2). This means that adult wet weight (which was used in our analyses of the effects of ant attendance) is a good indicator of dry weight in *A. fabae*. The equation for the regression of dry weight on wet weight was $y_{\text{dry}} = 0.236\,y_{\text{wet}}$ (Fig. 2), with a standard error of 0.005 for the slope.

### Colony growth

**Fig. 3:**
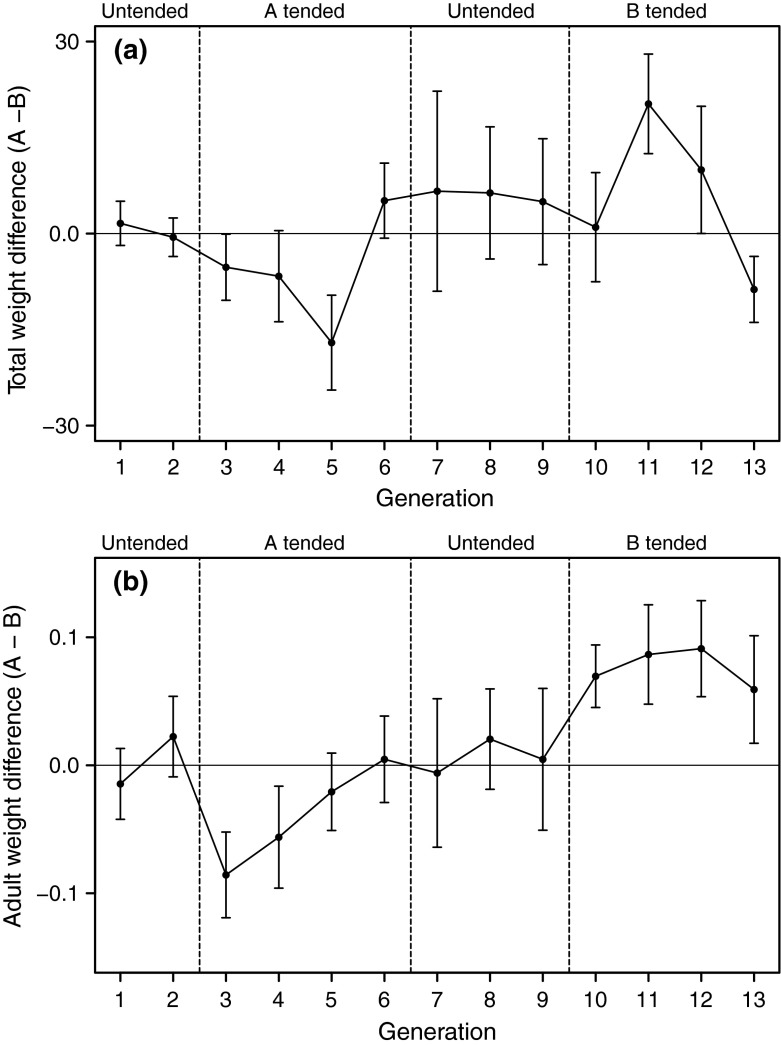
Total aphid colony weight difference (mean ± SE; mg) between paired cages (**a**) and the average adult weight difference (mean ± SE; mg) between paired cages (**b**) over 13 aphid generations. During the first two generations, all cages were untended; generations* 3*–*6*: cage A of each pair was ant tended (first ant treatment); generations* 7*–*9*: all cages were untended and during generation* 10*–*13* cage B of each pair was ant tended (reversal of the first ant treatment). Each* data point* is the average of the pairs present at that generation (there were 12 pairs at the start). *Dashed lines* indicate changes in treatment

There was a statistically significant effect of ant treatment on the difference in total aphid colony weight between the paired cages (Fig. 3; Table 1; over the experiment, the mean ± SD aphid colony weight was 48.8 ± 28.9 mg). Since the colony in each cage was restarted every generation using four adults from the previous generation in that cage, both the current ant treatment (lasting 1 week per generation) and the effect of the ant treatments in previous generations could in principle influence the colony weight. We found that, following the start of ant tending and over a period of a few generations, tended colonies weighed less when collected compared with untended colonies (Fig. 3). This effect, however, subsequently decreased and could no longer be detected after four generations of ant tending. The pattern was repeated in the second treatment period (generations 10–13; Fig. 3), with an initial reduction in the weight of tended colonies and a subsequent increase to the level of untended colonies. A similar pattern of changes was seen for average adult weight in the colony (Fig. 3).

**Table 1:** Bayesian statistical analysis of tended versus untended aphid weight differences, examining the effect of time (generations) during black garden ant *Lasius niger* treatment on total black bean aphid *Aphis fabae* colony weight difference, average adult weight difference, and total nymph weight difference between tended and untended cages in the pairs

Response variable	Parameter	Post. mean	95 % credible interval	*p*_MCMC_
Total weight	Intercept	−12.64	(−20.77, −4.22)	**0.005**
	*T* − *T*_0_	0.65	(−1.92, 3.18)	0.610
	(T − *T*_0_)^2^	3.63	(1.55, 5.84)	**0.001**
Adult weight	Intercept	−0.077	(−0.117, −0.036)	**0.001**
	*T* − *T*_0_	−0.004	(−0.023, 0.016)	0.670
	(T − *T*_0_)^2^	0.017	(0.003, 0.032)	**0.024**
Total nymph weight	Intercept	−6.75	(−11.31, −2.25)	**0.004**
	*T* − *T*_0_	0.38	(−1.08, 1.78)	0.600
	(T − *T*_0_)^2^	2.21	(1.05, 3.43)	**0.001**

**Fig. 4:**
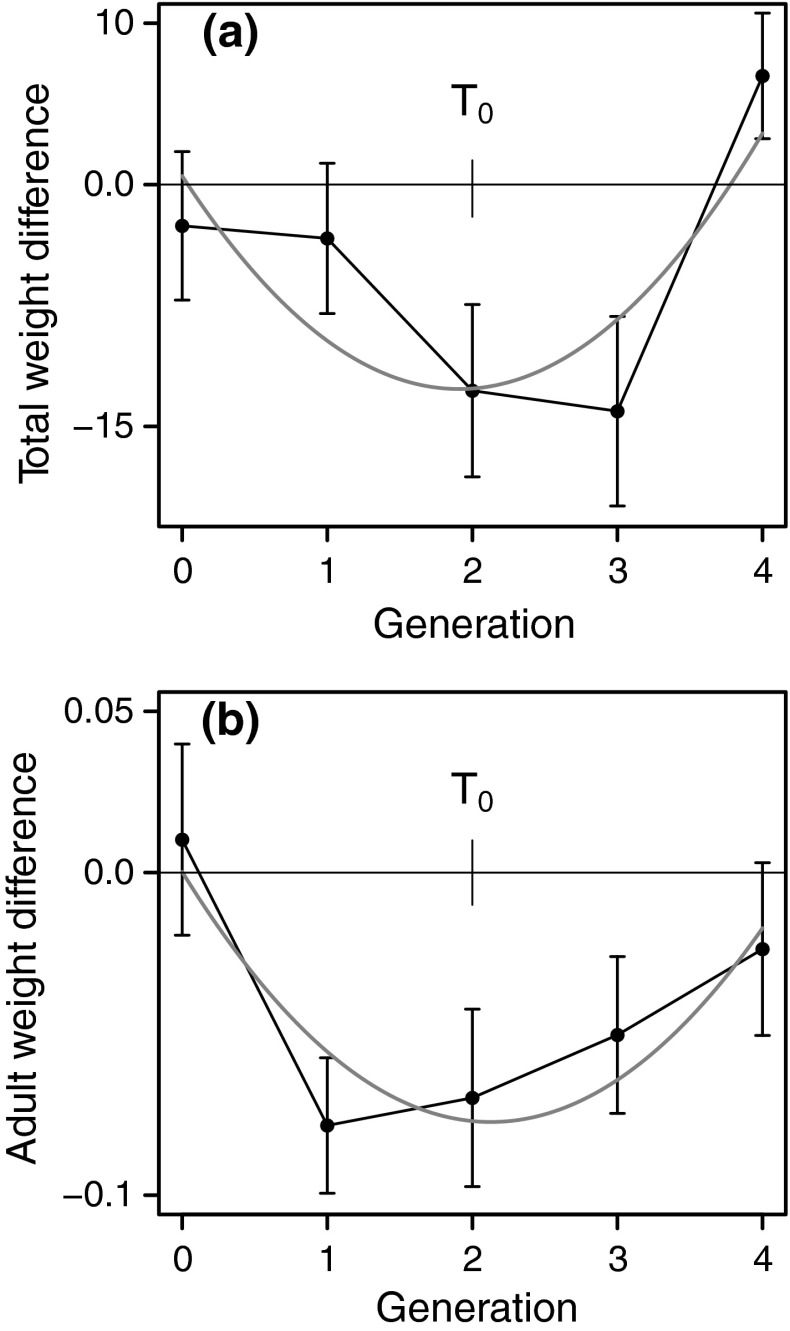
Observed and model fitted (*red line*; see Table 1) total aphid colony weight difference (**a**) and average adult weight difference (**b**) between tended and untended cages in pairs. The two ant treatment periods are analyzed together and time is measured such that generation 1 is the start of ant tending in each period. Data are given as mean ± SE (mg) for the pairs present at that time. There were 12 pairs at the start of the first ant treatment and 9 at the start of the reversed ant treatment. *T*_0_ is the generation used for the intercept in the model fitting (Table 1) (color figure online)

Mixed-effect models were fitted, with time expressed as the deviation of the generation *T* from *T*_0_, where *T*_0_ is the second generation of ant treatment (see Eq. (1) and Fig. 4)

See “Materials and methods” for description of the Bayesian MCMC analysis. As indicated by the smallest DIC, only a random effect for *a* in Eq. (1) was included in the analysis of total weigh difference and total nymph weight difference, whereas random effects for *a*, *b* and *c* were included for adult weight difference. The results did not depend on this selection of models: we found the same qualitative statistical significances regardless of whether only the first (intercept) or all tree random effects were included

Significant values shown in bold

For the total colony weight and the average adult weight, a Bayesian mixed model statistical analysis showed that the weights were significantly lower for the tended colonies at the end of the second generation after the start of ant tending (Table 1; Fig. 3). For the total colony weight and the average adult weight, there was also a statistically significant quadratic time dependence, with a minimum near the end of the second generation after the start of tending (Fig. 4; Table 1). The statistically significant quadratic time dependence of the tended–untended weight difference indicates the presence of transgenerational effects (Fig. 4); the weight difference did not only depend on ant tending in the current generation but was influenced by the previous history of tending. We found the same qualitative effect of ant tending on the total nymph weight (Table 1).

**Fig. 5:**
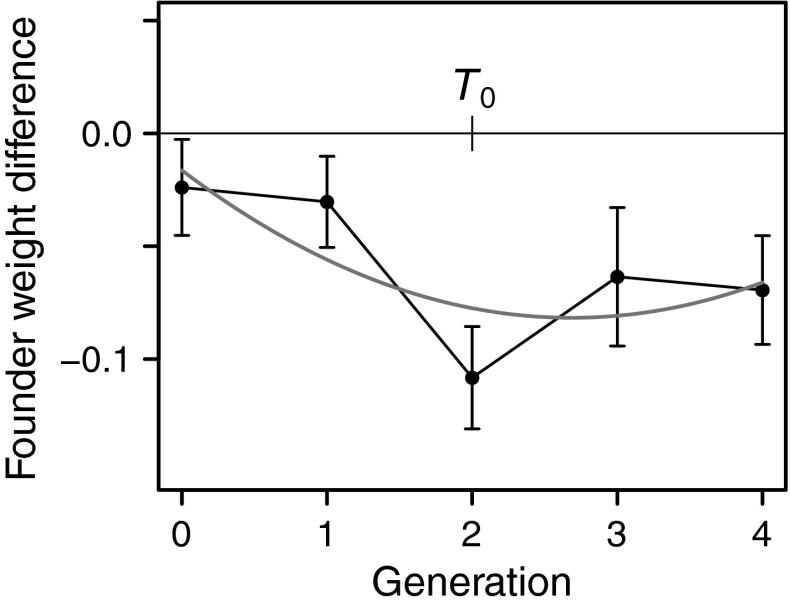
Observed and model fitted (*red line*) average founder weight difference between tended and untended cages in pairs. Data are given as mean ± SE (mg) for the pairs present at that time. The data analysis is the same as in Fig. 4 (color figure online)

The result of Bayesian fitting of a similar mixed model to the average founder weight is illustrated in Fig. 5 and showed that the founder weight of the tended colonies was significantly lower in the second generation after the start of ant tending (*p*_MCMC_ = 0.001). The temporal pattern (Fig. 5) suggests that the lowering of the average founder weight was shifted later by one generation compared with the average adult weight (Fig. 4b), consistent with the fact that the founders of a given generation were chosen from the adults of the previous generation.

### Effects of founder weight

The difference in founder weight from the second generation of ant tending and onwards led us to investigate whether average founder weight in itself influenced the development of a colony. We fitted a linear model to the average adult weight data from each cage and generation, controlling for the main effects of cage, generation and ant treatment, including the average founder weight as a covariate. There was no statistically significant effect of founder weight (*p* = 0.19), but there was an effect of ant treatment (*p* < 0.001). The estimated size of the effect of ant tending from the fitted model was 0.054 mg, which is in agreement with the average effect of ant treatment seen in Fig. 4b. Over the entire experiment, the average adult weight in collected colonies was 0.63 ± 0.13 mg (mean ± SD) and the average founder weight was 0.68 ± 0.14 mg, showing that the founders were slightly heavier. A similar analysis of total colony weight also showed no significant effect of average founder weight. We thus conclude that founder weight in itself was not directly responsible for the transgenerational effect of ant tending seen in Fig. 4.

### Embryo size distribution

**Fig. 6:**
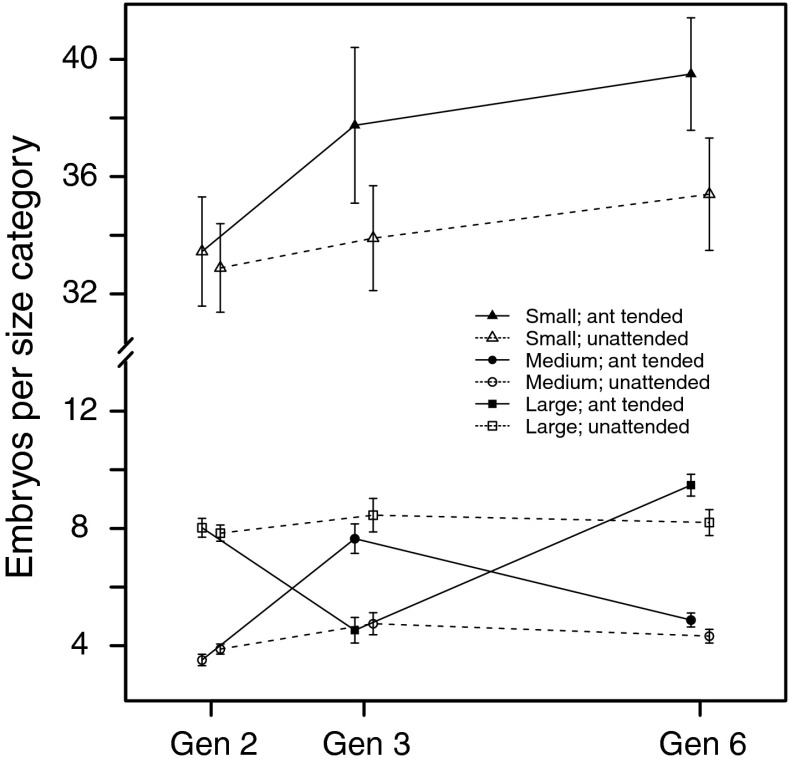
Number of large (length >0.05 mm), medium-sized (between 0.03 and 0.05 mm), and small (between 0.005 and 0.03 mm) embryos in dissected aphids, over the first part of the experiment (data shown as mean ± SE). During generation 2, all cages were untended; in generations 3–6, one cage of each pair was ant tended (first ant treatment). *Solid* (*dashed*) *lines* connect data points from cages that were ant tended (untended) in generations 3–6, and *filled* (*open*) *symbols* show the mean number of embryos of each size category in adult aphids that were tended (untended) during their life

Transgenerational effects of ant tending on aphid adult and colony weights could be caused by differences in reproductive investment between tended and untended aphids. To examine this possibility, we analyzed the size distribution of embryos in dissected apterous aphids at different points of time during the experiment: before ant tending (generation 2), in the first generation of ant tending (generation 3), and in the fourth generation of ant tending (generation 6). In generation 3, ant-tended aphids had fewer large but more medium-sized embryos compared with untended aphids (Table 2; Fig. 6), indicating a smaller reproductive investment in the tended aphids. In the fourth generation of ant tending (generation 6), this effect had disappeared, and to some extent had been reversed, with statistically significantly more large embryos in tended compare with untended aphids (Table 2; Fig. 6).

**Table 2:** Bayesian statistical analysis of the effect of ant tending on the number of embryos in different size categories, for generations 3 and 6 of the experiment (see Fig. 6)

Generation	Embryo size category	Effect	95 % credible interval	*p*_MCMC_
3	Large	−0.61	(−0.77, −0.43)	**0.001**
	Medium	0.42	(0.26, 0.59)	**0.001**
	Small	0.05	(−0.13, 0.23)	0.600
6	Large	0.15	(0.04, 0.27)	**0.020**
	Medium	0.11	(−0.02, 0.25)	0.124
	Small	0.12	(−0.01, 0.27)	0.094

See “Materials and methods” for description of the Bayesian MCMC analysis. The effect is the difference between tended and untended cages of the transformed variable log(*y*_c_ + 1), where *y*_c_ is the number of embryos in size category c, and generation 3 was the first ant tended generation

Significant results shown in bold

Examining the embryo size distribution in untended aphids, we did not find statistically significant changes between generations 2, 3, and 6 in the number of large, medium-sized, or small embryos (*p*_MCMC_ > 0.1 for all comparisons). For the number of small embryos, there was a fair amount of variation between aphids (Fig. 6), making it harder to detect differences between the generations.

### Bean plants with and without ants

The number of ants present on the bean plants of the ant-treated cages at the time the colonies were collected was 36.8 ± 16.2 (mean ± SD). Of these plants, only 5.2 % had fungus growing on them, whereas 72.0 % of plants collected from cages without ant treatment (on which no ants were found) had fungal growth, a statistically significant difference (*χ* ^2^ = 95.6, *df* = 1, *p* < 0.001).

## Discussion

Our study shows that *A. fabae* can modify its life-cycle strategy when tended by *L. niger*. Compared with previous work on this mutualism that examined costs of ant attendance in terms of reduced growth or slower development (El Ziady and Kennedy 1956; Banks 1958; El-Ziady 1960; Stadler and Dixon 1998), our results agree with those of Stadler and Dixon (1998) and Yao et al. (2000) in finding a cost for aphids in reduced colony growth, which the other studies did not find. The changes over the generations in the embryo size distribution in ant-tended aphids followed the same qualitative pattern as the adult aphid weight, indicating that at least part of the effect of ants on aphid total colony and adult weight was mediated through a change in the aphid reproductive investment. Thus, in generation 2 of the experiment, before ant tending was first introduced, there were around eight large embryos and four medium-sized embryos in an adult aphid. In the first generation that experienced ant tending, the distribution of embryo sizes changed markedly, with only four large but now eight medium-sized embryos per tended aphid. In the fourth generation of ant tending, however, the embryo size distribution had become similar to that before the contact with ants (there was even an increase in the number of large embryos; Fig. 6).

Our embryo size categories are based on embryo length. Investment of resources into embryos is likely to be proportional to embryo volume, which can be approximated as proportional to the cube of the length. Considering our size categories, it then follows that a large embryo, say having a length of 0.055 mm, has around 2.6 times the volume of a medium-sized embryo, say having a length of 0.04 mm. This means that the changes in embryo size distribution we observed in connection with ant tending correspond to quite substantial differences in reproductive investment.

As the number of ovarioles is fixed in the parthenogenetic phase (Dixon and Dharma 1980b), altering the embryo size distribution may be the only way for the adult aphid to change its reproductive strategy when circumstances in the environment change. In general, large individuals tend to produce larger and more offspring as adults and start reproducing earlier than small aphids (Dixon and Dharma 1980a). A change in the embryo size distribution towards smaller sizes, which we observed in the first generation of ant-tended aphids, is thus likely to be associated with smaller and/or fewer offspring and possibly also delayed reproduction. In the same way, the reproductive capacity of the aphids in the fourth generation of ant tending are likely to have been the same or even greater than that of untended aphids.

Around two generations after the start of ant tending in our experiment, we measured a notable decrease in aphid colony weight: the weight at the time of collection was reduced by about 25 % in ant-tended colonies (Figs. 3, 4), indicating a cost of ant attendance. It is of course not straightforward to compare the cost of mutualism between systems, but our estimate for the aphid–ant interaction is within the range found for other mutualisms (Bronstein 2001). Further, our results are the first to demonstrate how the aphid response to ant tending changes over several generations. The cost, or investment, expressed as reduced colony growth, initially increased over two aphid generations, but then decreased and could no longer be detected in the fourth generation of interaction with ants. The effects of ants included a reduction in the average adult aphid weight (Figs. 3, 4), which implied a subsequent reduced founder weight (Fig. 5), but the weight of the colony founders per se did not appear to be a major cause of the change over time of the effect of ant interaction. Hence, it appears that interaction with ants can trigger phenotypic changes in aphids that go beyond immediate behavior, such as droplet delivery rate, and can be passed on to offspring. Changes in the embryo size distribution (Fig. 6) could play a role in mediating these effects.

Because ants do not forcibly extract honeydew from aphids, but rather collect what the aphids deliver, it is reasonable to assume that the costs associated with ant tending derive from aphid investments that serve to modify ant behavior in a way that is beneficial to the aphids, at least in certain situations. One possibility is that aphids initially invest in establishing an interaction with ants, by inducing the ants to collect honeydew at their location. Ants are also able to relocate aphids and somehow judge host plant quality and increase settlement on nearby high-quality hosts (Collins and Leather 2002). Aphids, like other trophobionts, compete for ant attendance with other food sources, including other trophobionts and extrafloral nectaries (Cushman and Addicott 1989; Del-Claro and Oliveira 1993), so they may need to increase their attractiveness in order to ensure a sustained foraging response by the ants. Another possibility is that the investment acts as an appeasement that protects aphids against ant predation (Offenberg 2001; Oliver et al. 2009). In particular, the initial ant–aphid contact might involve a higher risk of ant attack (Glinwood et al. 2003), in which case a higher rate of honeydew release by the aphids could be part of a defense response. As aphids are tended by ants, they get covered in cuticular hydrocarbons which inform the ants that these aphids have been previously tended by their colony, tended by other colony, or that they are untended (Endo and Itino 2012). Untended aphids have a higher risk of ant predation, and aphids tended by ants from the same colony suffer the lowest risk of being predated.

The reduction, or even elimination, of the cost of ant interaction after four generations may reflect the fact that the ant-tended aphids in our study experienced a reliable tending and no attacks by natural enemies, leading to a decrease in investment and cost over time. In general, aphid investment in ants may be expected to respond to various cues that indicate the willingness of ants to interact and the risk and seriousness of natural enemy attack. For instance, competition between inter- and intraspecific aphid colonies can influence aphid survival (Cushman and Addicott 1989), illustrating the importance for aphids of being sufficiently attractive to ants. The protection provided by ants might also be especially beneficial in certain phases of the aphid life cycle, such as the initial growth phase of an aphid colony, when the colony is small and vulnerable. Small colonies in the field have been found to have a higher probability of persisting and growing when tended by ants (Breton and Addicott 1992), and colony survival is positively correlated with the number of tending ants. There may of course be circumstances where the presence of ants is harmful to the aphids. For instance, parasitoid attacks have been observed to increase when aphids receive ant attendance (Völkl 1992; Kaneko 2003; Mondor et al. 2008), perhaps because parasitoids can use ants as a cue to locate aphids and benefit from the protection afforded to their developing larvae inside tended aphid colonies (Tegelaar et al. 2012).

We found that the presence of ants greatly reduced fungal growth on aphid-infested bean plants. This was most likely due to efficient collection of delivered droplets and cleaning of honeydew from the leaves by ants. Both aphids and host plants might benefit from this, because fungal growth can damage the growth of aphid-infested plants by reducing light uptake and increasing the amount of necrotic tissue (Rabbinge et al. 1984; Dik et al. 1991). This is consistent with plant-increased extrafloral nectar production attracting ants upon aphid infestation (Jaber and Vidal 2009). In our study, the level of fungus infestation was low on bean plants due to short infestation periods and a change of host plant each generation, which reduced the risk that differences in host plant quality, such as fungus infestation, might cause systematic changes in reproductive investments over successive generations.

Phenotypic plasticity in aphids is sometimes controlled by a combination of photoperiod, crowding, and predator cues (Agarwala 2007), but for reproductive investments, the mechanisms of plasticity are not known. In our study, the photoperiod, crowding, and predator cues were controlled in the experimental set-up. Concerning the changes in aphid reproductive strategies, it could be that endocrine control of reproduction, e.g., via a physiological mechanism that responds to pheromones from the ants, similar to what has previously been found to control phenotypic plasticity in aphids, plays a part in explaining our results. In general, in insect phenotypic plasticity, hormones have been found to be linked to changes in environmental factors, such as temperature, photoperiod, and crowding (Nijhout 1999; Hartfelder and Emlen 2012).

Based on our observations and the work by Stadler and Dixon (1998), it appears that the interaction between *A. fabae* and *L. niger* is a case of pseudo-reciprocity (Connor 1995; Leimar and Hammerstein 2010), where the aphids at least to some extent make costly investments to obtain by-product benefits in the form of the ant tending behavior or reduced predation by ants. Whether these alterations of reproductive strategy are aphid-controlled or induced by ant manipulation cannot yet be decided. A general conclusion that emerges from our work is that aphids may be similar to many other trophobionts in showing flexibility in the investment in ant rewards (Leimar and Axén 1993; Axén et al. 1996; Axén and Pierce 1998; Agrawal and Fordyce 2000; Morales et al. 2008), and it should be expected that aphid investments in ant tending respond to factors such as changes in the perceived risk of enemy attack. A novel aspect of our work is that it suggests a role for transgenerational effects in this kind of flexibility.

## Electronic supplementary material

Below is the link to the electronic supplementary material.

Supplementary material 1 (PDF 828 kb)

Supplementary material 2 (ZIP 6 kb)
