# Supplementary material for: Transgenerational effects and the cost of ant tending in aphids
Source: Oecologia. 2013 May 21;173(3):779–90. doi: 10.1007/s00442-013-2659-y (PMC3825118; doi:10.1007/s00442-013-2659-y)
Supplement: Supplementary file 1 — Supplementary material 1 (PDF 828 kb) [file 442_2013_2659_MOESM1_ESM.pdf]

a)

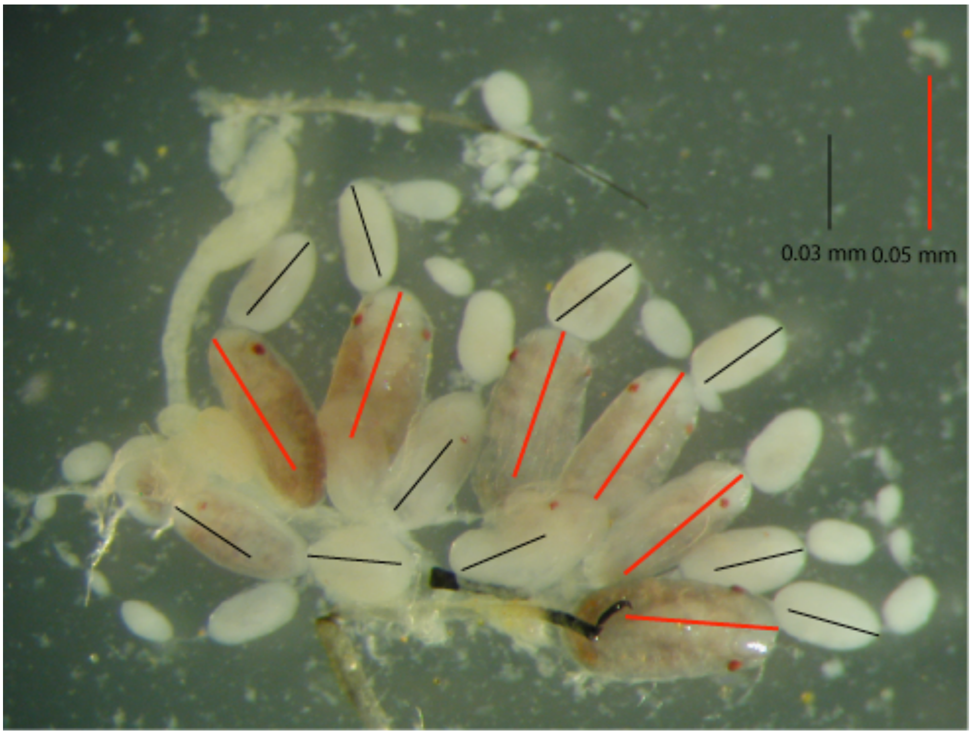


b)

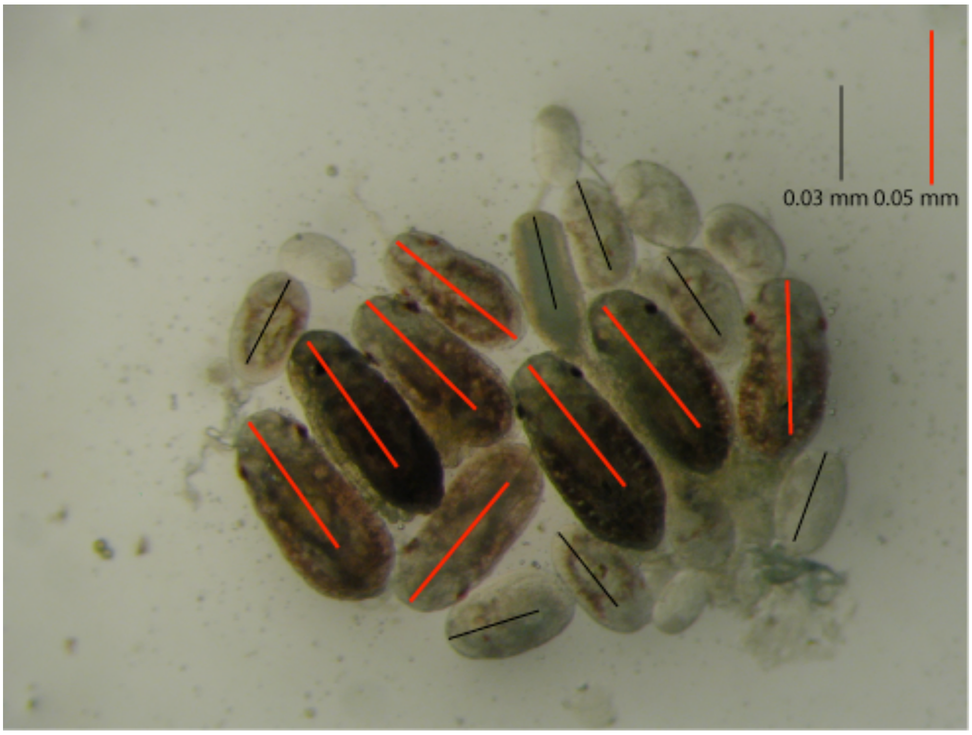


**Figure S1.** (a) Embryos from one adult aphid dissected directly after euthanization, and (b) embryos from one adult aphid, preserved in 70% ethanol for 3 days. Notice the darker color of the embryos in (b) compared to (a), which is caused by dissolved aphid cuticular pigments that become deposited in the tissues of the embryos. This process makes it easy to count and measure also the smaller embryos. The large embryos are indicated by red line segments (0.05 mm in length), and the medium sized embryos are indicated by black line segments (0.03 mm in length). The numbers of large and medium sized embryos differ between the two aphids with 6 large and 10 medium in (a) and 8 large and 7 medium in (b).
